# Determination of Heat Transfer Coefficient for Air-Atomized Water Spray Cooling and Its Application in Modeling of Thermomechanical Controlled Processing of Die Forgings

**DOI:** 10.3390/ma15072366

**Published:** 2022-03-23

**Authors:** Marcin Apostoł, Piotr Skubisz, Henryk Adrian

**Affiliations:** 1 Faculty of Mechanical Engineering and Robotics, AGH University of Science and Technology in Cracow, 30 Mickiewicz Avenue, 30-059 Cracow, Poland; 2 Faculty of Metals Engineering and Industrial Computer Science, AGH University of Science and Technology in Cracow, 30 Mickiewicz Avenue, 30-059 Cracow, Poland; pskubisz@agh.edu.pl (P.S.); adrian@agh.edu.pl (H.A.)

**Keywords:** thermomechanical controlled processing, heat transfer coefficient, atomized water spray cooling, inverse heat conduction problem, hot forging, direct cooling

## Abstract

The paper presents the method and results of determination of heat transfer coefficient for air-atomized water spray cooling with consideration of infrastructural factors of industrial cooling conveyor, such as effect of accelerated air. The established values of heat transfer coefficient were implemented into a numerical model of cooling line, with special definition of sprayers and the movement of the part subjected to quenching. After quantitative validation on selected samples, the obtained coefficients were used for the solution of the technological problem by means of localized cooling rate enhancement, which forms a case study confirming reliability of the established water spray heat transfer functions and suitability of the determined models for design of thermomechanical controlled processing of complex-geometry parts.

## 1. Introduction

There are many fields of application of spray technique in processing of metals and alloys. Development of controlling systems and easiness of integration and synchronization of complex multi-jet systems are major factors inducing wide use of atomized spray in numerous aspects of materials technologies. Beside crystallization of continuous cast slab, cleaning, and descaling [1,2,3], air-atomized water spray finds its application among cooling practices used in heat treatment or thermomechanical treatment of, mostly, but not only, steel products [4,5,6].

Thermomechanical processing of steel to a great extent depends on controlled cooling. Desired fractions of overcooled austenite products, which are the base for multiple-phase steel and/or precipitation kinetics controlling strengthening effect as well as solution treatment efficiency, depend on accommodating a proper rate and course of cooling in time. Therefore, beside bath or stream of water for direct quenching or fan cooling for obtaining of ferrite-pearlite microstructures from overcooled austenite, air-atomized water spray is often used.

Specific features and characteristics of atomized water–air spray are reasons for its use in heat treating operations of metal formed components without restriction on size or materials. Spray cascades are effective in cooling long profiles in continuous stationary processes, e.g., rolled products [5,7,8]. It works well while cooling thicker sections, such as forged shafts or tubes [9,10], where increased cooling rates in relation to accelerated air are necessary, yet on the other hand, prevent excessive overcooling, which might lead to overquenching the surface and/or excessive stresses and distortion. It makes a good solution where bath cooling offers a too high cooling rate, high infrastructure expenses and, in addition to this, limited versatility and incapability of locally diversified heat transfer [11].

In the practice of thermomechanical controlled processing, spray cooling enables direct quenching of forged parts directly from hot deformation temperatures [8,12] and is a convenient way for realization of interrupted cooling [13]. Technological realization of continuous cooling with interrupted quenching, isothermal quenching, austempering or martempering and tempforming [14,15], and last but not least, heat treating large cross-sections calls for instant intensified heat transfer conditions with simultaneous flexibility of controlling heat flux for an optimized run of cooling curve in crucial locations in relation to critical points and/or time intervals to provide proper kinetics of nucleation of microstructure strengthening/controlling precipitates [16,17,18,19,20].

Application of atomized air spray for realization of heat treating offers the possibility of a control heat transfer rate by adjustment of jet impingement characteristics by setting water–air proportion, size and distribution of droplets, flow rate and pressure. As compared to water lathes atomized air spray allows for more uniform flux characteristics and distribution of flux over the surface. In addition to this, effect of the geometry of nozzle and orientation of atomizers on heat transfer efficiency and other geometrical considerations [21,22,23,24] enable selection of atomizer types for the extension of the cooling area and for the continuous cooling processes, e.g., cooling conveyors, control of the cooling time.

Characteristics of atomizers are widely accessible, which makes it easy to select ones proper for the needs, and determine their parameters for the desired effect [10,23,25,26,27,28]. However, many of the available results are sensitive for case and the need of scaling up come up to include environmental considerations. Such cases are likely in specialized cooling lines, for instance, in, technological realization of interrupted quenching as implemented in a continuous cooling process, which involve combination of water spray cooling with fan-accelerated air. Then, relying on literature data may be insufficient. Mutual interaction of the both media creates a mixed environment and the necessity of a mathematical model of heat transfer conditions.

Similarly to many related studies, as an alternative for mathematical modeling based on phenomenology, numerical methods based on the solution of an inverse problem is used for definition of the heat transfer coefficient [27,29,30,31]. The goal of this paper is formulation of heat transfer coefficient (HTC) function for mixed environment of air-atomized water spray simultaneously with accelerated air and its use in modeling of interrupted controlled cooling, which forms a case study illustrating the application of the results in forging practice.

## 2. Models and Assumptions

### 2.1. Experimental Procedure

The goal of this work was definition of the heat exchange model and elaboration of the temperature dependence of the heat transfer coefficient for air-atomized water spray combined with fan-accelerated air to use it for design of thermomechanical treatment cycles. Experimental measurements and validation tests were conducted in laboratory conditions on the simulator Quench Tube Lab, a full-scale model of industrial cooling conveyor, and equipped with location, temperature, pressure and air rate sensors enabling all necessary measurements. Dependent on the needs, the laboratory stand enabled reciprocal passage through consecutive zones or stationary cooling to simulate production-like conditions. In the study, both of these two modes were used, 1—for estimation of heat transfer coefficient, and 2—for controlled cooling tests.

The analysis of the effect of the cooling parameters on the heat transfer coefficient, α, was carried out in the laboratory stand Quench Tube. The apparatus and the method of measurement of cooling rate is described in [31]. Recorded relationships between the temperature inside the test probe, *T_s_*, and the cooling time, *τ*, were used for calculating the relationships between the heat transfer coefficient, α, and the surface temperature, *T_p_*, by the numerical solution of the inverse problem of heat conduction equation using Weber method [32]. The relationships between cooling rate, *v_c_*, and temperature, *T*, were also calculated.

**Figure 1:**
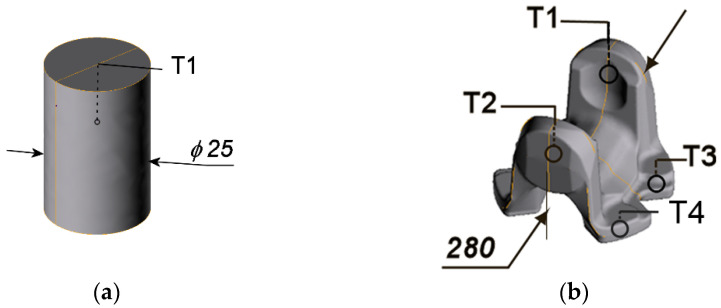
Geometry of samples used in the study: (**a**,**b**) samples used for validation of the estimated heat transfer coefficient, where T1 ÷ T4 are designations of thermocouples.

In order to validate the obtained functions of heat transfer coefficient, *α =* *f(T_p_)*, cooling tests were carried out. Two sample geometries were used for this purpose: (1) cylindrical sample testing samples of diameter 25 mm and height 37 mm (total volume of the sample embraced by spray) made of martensitic stainless steel X40Cr13 (AISI 420) so as to get rid of transformation latent heat effects, shown in Figure 1a, and sample (2) complex-geometry forged part of significant weight made of medium carbon steel, shown in Figure 1b, (selected to verify the determined characteristic in case of partial covering the part by spray), results of which are initial effect of heat treatment modeling.

Determination of heat transfer coefficient functions involved settings (air/water pressure, velocity, configuration, etc.) reasonable from the standpoint of their utilization in cooling cycles designed for forged cardan in the continuous cooling line. Thus, a series of combinations of spray inlet(s) with or without action of accelerated air was selected, that is: upper central spray only, upper central spray plus accelerated air, upper central plus bottom central, upper slant, two sided symmetrical spray inlets and, in addition to this, spray plus air at conveyor movement.

The cooling conditions occurring in the cooling zone, correspondingly to conditions expected during cooling tests, are given in Table 1.

**Figure 2:**
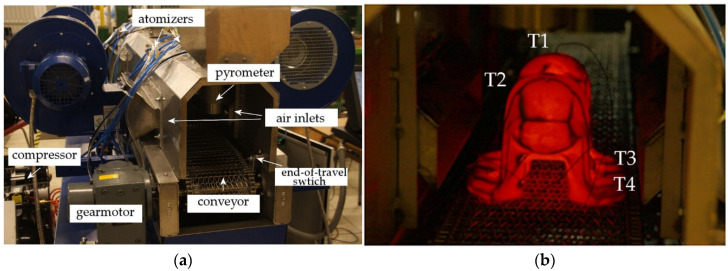
Experimental cooling tests: (**a**) controlled cooling simulator QuenchTube Lab, (**b**) cooling of the forged sample used for the measurements.

Having confirmed an agreement between the experiment and numerical simulation, the second part of the study was conducted—estimation of the process conditions producing favorable course of cooling curves from the standpoint of transformation kinetics on cooling. It involved experimental tests of controlled cooling (Figure 2), aided with finite element method (FEM) simulation in aspects of varying and optimizing the cooling conditions. For this purpose, the cooling simulator was implemented into the technological chain of a forge plant to attain strain and temperature history from the forging process. The FEM analysis included shape progression during a sequence of forging stages and subsequent transformation of austenite on cooling, including post-processing calculation of temperature and volume fractions of structural components in selected locations. The conditions assumed in heat transfer measurements were based on fluid flow simulation results (Section 3.1) with consideration of symmetrical and asymmetrical placement of atomizers. Thus, a set of five combinations of settings was selected for further analysis, as shown in Table 2.

### 2.2. Solution of the Inverse Heat Conduction Problem

In the boundary inverse heat conduction problem heat flux density on the surface and the heat transfer coefficient on the edge of the heated(cooled) object are under consideration. The inverse problems are ill-posed, that is, a small disturbance on the input is transferred to the increasing errors of the output results due to sensitivity to the measurement errors. Thus, appropriate method to mitigate the effects of poor conditions of the problem is necessary in order to obtain correct results of the inverse problem solution. Measured temperature records may contain errors related to the occurrence of electronic noise during the recording electric signal from thermocouple. Their impact can be reduced using electronic noise reduction techniques.

The purpose of the modification methods for solving inverse problems with improved stability of the calculations results, modification methods have been developed. One group of the methods which find their application in solving the parabolic boundary inverse heat conduction problem are space marching methods. One of the algorithms used in these methods, based on the solution of a linear one-dimensional inverse problem, was developed by Weber [32]. It involves replacing the parabolic differential equation of heat conduction by the hyperbolic equation. This approximation makes the problem well-posed. Directly measured temperature data are used here, thus the hyperbolic approximation of the inverse heat conducted problem may be insufficient to eliminate high noise input data. Modified heat conduction equation in a cylinder coordinates’ system with one-way flow of heat (system 1D) can be formulated as follows:

(1) $$\beta \rho (T)c_{p}(T)\frac{\partial^{2}T(r,t)}{\partial t^{2}}+\rho (T)c_{p}(T)\frac{\partial T(r,t)}{\partial t}=\frac{1}{r}\frac{\partial }{\partial r}\left(r\lambda (T)\frac{\partial T(r,T)}{\partial r}\right)$$

with the initial condition:

(2) $$T(r,0)=T_{o},\;0<r<R$$

and the following boundary conditions:

(3) $$\frac{\partial T(r,t)}{\partial r}=0\;\mathrm{at}\;r=0\;\mathrm{and}\;t\;>\;0$$

Heat flux *q*:

(4) $$q(r,t)=-\lambda \frac{\partial T(r,t)}{\partial r}$$

is to be estimated at *r* = *R* and *t* > 0.

The additional condition for solving the inverse problem is:

(5) $$T(0,t_{k})=T_{measured}(t_{k})\;\mathrm{for}\;r=0\;\mathrm{and}\;k=1,2\ldots k$$

To solve this equation using a finite difference method, FDM, a symmetric difference scheme to approximate the temperature of the partial derivatives of the second order with respect to time and location was applied, and the forward difference scheme symmetrical front to approximate the first derivative of temperature with respect to time and a symmetrical difference scheme with respect to position [33]. The finite difference form of Equation (1) is:

(6) $$\frac{\beta \rho c_{p}\Delta r}{\Delta t^{2}}\left[T_{i}^{k+1}-2T_{i}^{k}+T_{i}^{k-1}\right]+\frac{\rho c_{p}\Delta r}{2\Delta t}\left[T_{i}^{k+1}-T_{i}^{k-1}\right]=\frac{\lambda_{i}}{\Delta r}\left[T_{i+1}^{k}-T_{i}^{k}\right]-\frac{r_{i-1}\lambda_{i-1}}{r_{i}\Delta r}\left[T_{i}^{k}-T_{i-1}^{k}\right]$$

where subscript *i* is a grid space number, and *k*—time index.

For *i* = 1:

(7) $$T_{i+1}^{k}=T_{i}^{k}+\frac{\beta \Delta r^{2}}{a_{i}^{k}\Delta t^{2}}\left[T_{i}^{k+1}-2T_{i}^{k}+T_{i}^{k-1}\right]+\frac{\Delta r^{2}}{2a_{i}^{k}\Delta t}\left[T_{i}^{k+1}-T_{i}^{k-1}\right]$$

for, 1 < *i* < *n_p_*.:

(8) $$T_{i+1}^{k}=T_{i}^{k}+\frac{\beta \Delta r^{2}}{a_{i}^{k}\Delta t^{2}}\left[T_{i}^{k+1}-2T_{i}^{k}+T_{i}^{k-1}\right]+\frac{\Delta r^{2}}{2a_{i}^{k}\Delta t}\left[T_{i}^{k+1}-T_{i}^{k-1}\right]+\frac{r_{i-1}\lambda_{i-1}}{r_{i}\lambda_{i}}\left[T_{i}^{k}-T_{i-1}^{k}\right]$$

for *i* = *n_p_*.:

(9) $$\frac{\beta \rho c_{p}\Delta r}{\Delta t^{2}}\left[T_{i}^{k+1}-2T_{i}^{k}+T_{i}^{k-1}\right]+\frac{\rho c_{p}\Delta r}{2\Delta t}\left[T_{i}^{k+1}-T_{i}^{k-1}\right]=\frac{1}{r_{i}}\left[-r_{i}q_{i}-\frac{r_{i-1}\lambda_{i-1}}{\Delta r}\left[T_{i}^{k}-T_{i-1}^{k}\right]\right]$$

The heat flux is calculated:

(10) $$q_{i}^{k}=\left[-\frac{r_{i-1}\lambda_{i-1}}{r_{i}\Delta r}\left[T_{i}^{k}-T_{i-1}^{k}\right]\right]-\frac{\beta \rho c_{p}\Delta r}{\Delta t^{2}}\left[T_{i}^{k+1}-2T_{i}^{k}+T_{i}^{k-1}\right]-\frac{\rho c_{p}\Delta r}{2\Delta t}\left[T_{i}^{k+1}-T_{i}^{k-1}\right]$$

Finally, heat transfer coefficient, *α*, is calculated:

(11) $$\alpha_{n_{p}}^{k}=\frac{q_{n_{p}}^{k}}{T_{\infty }-T_{n_{p}}^{k}}$$

For minimization of inaccuracy of the input temperature measurement, a combination of the hiperbolic approximation with Savitzky–Golay filter was used, as suggested by data Al-Khalidy [34,35,36]. Thus, average values of temperature in point *i*, for time step, *k*, could be calculated from equation:

(12) $$T_{i,k}=-0.086T_{i}^{k-2}+0.343T_{i}^{k-1}+0.486T_{i}^{k}+0.343T_{i}^{k+1}-0.086T_{i}^{k+2}$$

### 2.3. Assumptions and Boundary Conditions in FEM Modeling

Numerical modeling was conducted by means of the commercial program QForm3D v.10, based on finite element method (FEM) with use of Voronoi cell algorithm, used in advanced multistage hot forging and microstructure transformation simulation [37,38,39,40].

The metal flow simulation involves thermal–mechanical coupled analysis, based on assumption of viscoplastic model of incompressible deformed body and Levanov friction model [41,42], with friction factor 0.4. Forging simulation involves a kinematic model of mechanical press 25 MN, with working speed 40 stroke/min. Flow stress of work material, steel 38MnSV6 is described with Formula (13), with coefficients, determined by multiple regression of experimental data from uni-axial compression test, shown in Table 3.

(13) σp=Aem1TTm9εm2em4ε(1+ε)m5Tem7εε˙m3ε˙m8T

The system of equations governing computations of the metal flow mechanics and heat transfer in worked metal and tools can be found in [43].

**Figure 3:**
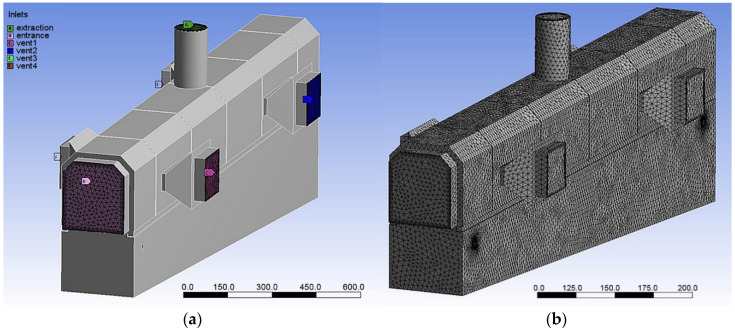
Numerical models in the flow simulation: (**a**) CAD-based model of cooling chamber of the laboratory cooling simulator, and (**b**) finite element mesh.

Thermal analysis was based on the determined functions of heat transfer coefficient for given settings of the cooling line (presented in Section 3.2). For natural air cooling (transfer and the end of the process) constant value 30 W/m^2^·K, water temperature 25 °C and temperature-dependent emissivity function based on [44] were assumed. As in the experiment where a one-sided or symmetrical air inlet could be alternatively used, both symmetrical and asymmetrical cooling conditions were taken into consideration. To account for the local difference in cooling conditions, numerical modeling of accelerated air cooling required a special domain to define *α* coefficient for exposed and shadowed surfaces. The assumption of heat transfer coefficient in these domains and the considered settings combinations (Table 1) was based on air-rate measurements and flow simulation so as to predict fluid behavior in the conveyor and air rate distribution for technically justified combinations of working elements (presented in Section 3.1). The flow simulation was conducted in ANSYS code. The numerical model was based on CAD geometry of the cooling chamber of the laboratory simulator Quench Tube (Figure 3a) with finite element mesh consisting of 250,441 nodes and 1,020,512 elements (Figure 3b).

**Figure 4:**
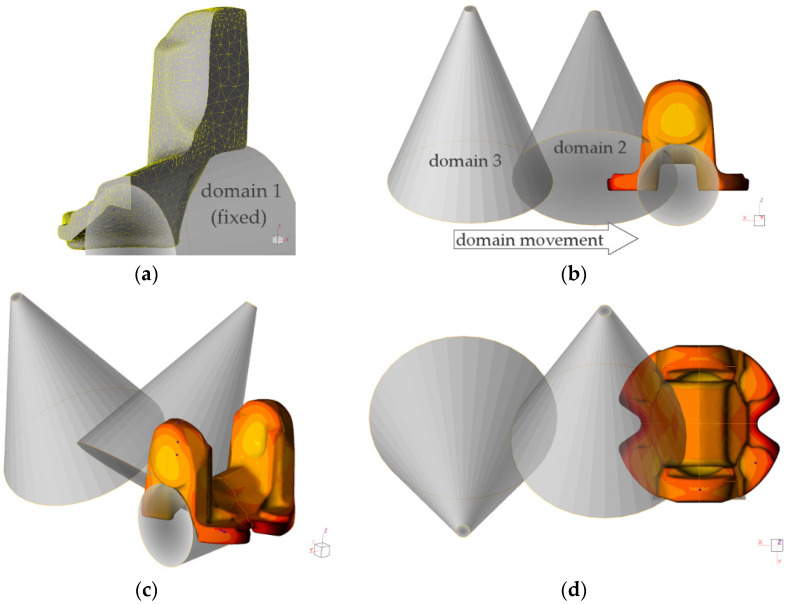
Numerical model of sprayer domains used in the simulation with definition of the established heat transfer coefficients (domains 2 and 3) and shadow area (domain 1), where: (**a**) numerical model of the forged part with tetrahedral mesh, (**b**–**d**) configuration of domains in front, isometric and top view, respectively.

In order to reproduce the actual cooling conditions, a sequence of eight stages was composed, i.e., (1) transfer from the forging press to the cooling line, (2) cooling in accelerated air of approximate rate 15 m/s, in which sprayer domains with the previously determined *α* coefficient were used (Figure 4), passing over the sample with speed of conveyor (Figure 4b), thereby forming three periods of spray quenching with intervals of fan-cooling acting on surfaces outside the sprayers (Figure 4c,d), followed by natural air cooling. The experimental plots of temperature measured in selected points of the part are presented in Section 3.2 when compared to those calculated numerically for thermocouples T1 and T3 (as the plot from thermocouple T1 was the same as T2 and similarly, T3 and T4; and any deviations were attributed to time shift during cooling, one of each pair is representative for corresponding areas by the symmetry).

## 3. Results

### 3.1. Flow Simulation

**Figure 5:**
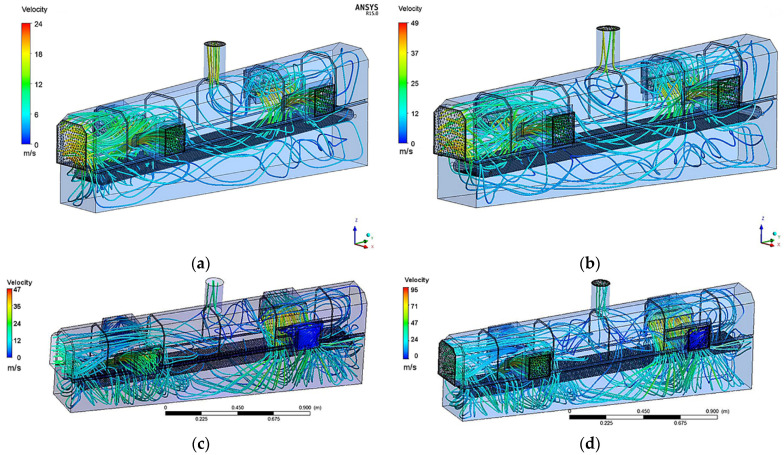
Results of numerical modeling of fluid flow stream lines in the cooling chamber for different setting of air rate: (**a**) 10 m/s—four vents, (**b**) 20 m/s—four vents, (**c**) 10 m/s—2 vents asymmetrical, and (**d**) 20 m/s—2 vents asymmetrical.

The number and configuration of active vents, as well as air rate let into the cooling zone of the conveyor, results in fluid streams flow and, eventually, distribution of heat transfer conditions. In addition to generating turbulent flow, increasing air rate has an impact on heat transfer coefficient [31]. As the fluid flow simulation shows, it changes in location on the length of cooling sections (Figure 5). In addition to this, air rate affects the mode of the flow from laminar to turbulent, which is obviously dependent on configuration of vents, which can be concluded from asymmetrical vs. symmetrical air inlet stream comparison. It was also observed that air rate produced on preset vents has risen at the exit of inlet. Thus, preset 10 m/s resulted in averaged 15–18 m/s and 20 m/s brought about averaged 25 m/s, which were taken for in further tests (Table 1).

### 3.2. Heat Transfer Coefficient

**Figure 6:**
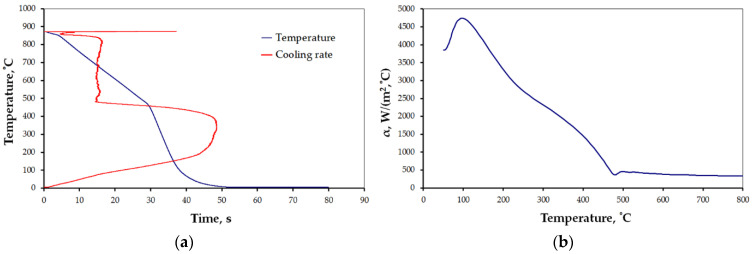
Results of measurement: (**a**) *T = f(τ)* and *v = f(T)*, and (**b**) heat transfer coefficient for settings 1 (Table 1).

**Figure 7:**
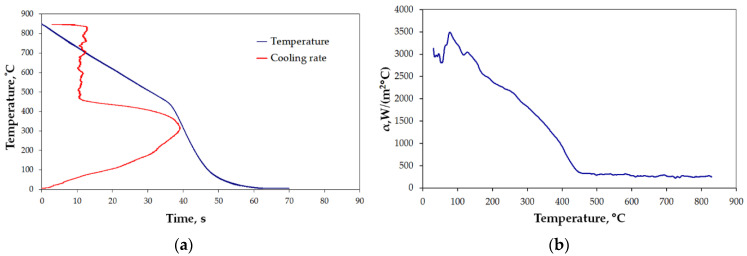
Results of measurement: (**a**) *T = f(τ)* and *v = f(T)*, and (**b**) heat transfer coefficient for settings 2 (Table 1).

**Figure 8:**
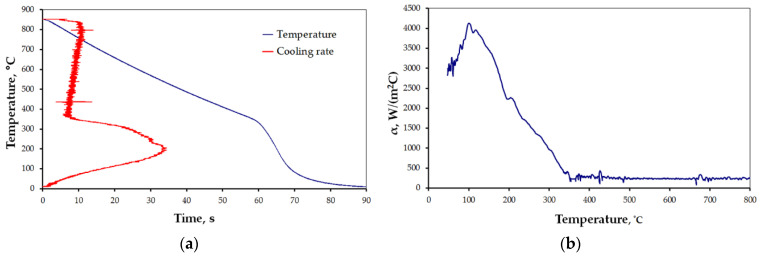
Results of measurement: (**a**) *T = f(τ)* and *v = f(T)*, and (**b**) heat transfer coefficient for settings 3 (Table 1).

**Figure 9:**
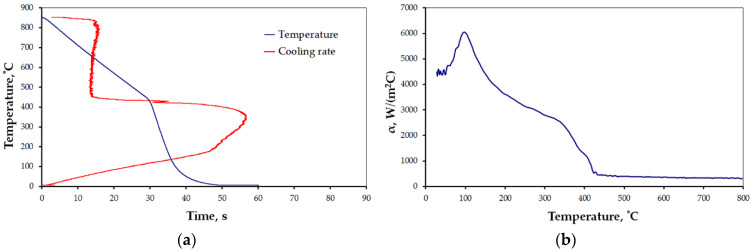
Results of measurement: (**a**) *T = f(τ)* and *v = f(T)*, and (**b**) heat transfer coefficient for settings 4 (Table 1).

**Figure 10:**
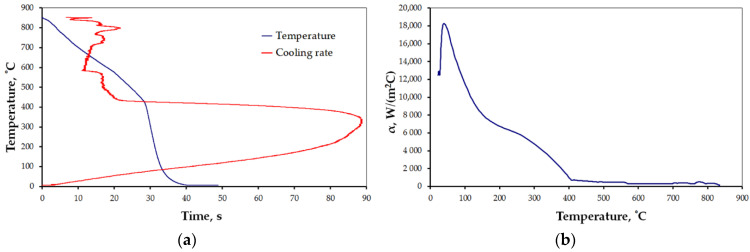
Results of measurement: (**a**) *T = f(τ)* and *v = f(T)*, and (**b**) heat transfer coefficient for settings 5 (Table 1).

**Figure 11:**
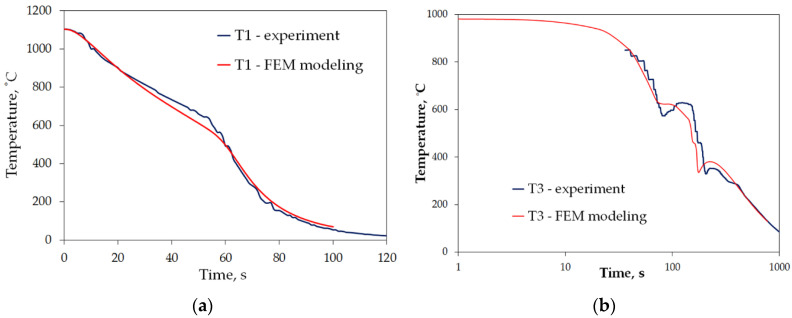
Validation of obtained heat transfer coefficients in cooling test: (**a**) small sample of martensitic stainless steel (Figure 1a), and (**b**) big sample—a complex-geometry forged part (Figure 1b).

The results for the selected cases are presented below (Figure 6, Figure 7, Figure 8, Figure 9, Figure 10 and Figure 11), where Figures “a” show original measurements of temperature inside the test probe versus time, *T = f(τ)* and their derivative—cooling rate (in °C/s), *v = f(T)*, and Figures “b”—plots of heat transfer coefficient, *α*, as a function of surface temperature *T*.

The shape of cooling curves (relationships *T = f(τ)*) is similar to curves obtained for liquid quenching media, with three stages, reflecting different cooling mechanisms during the quenching of hot metals: vapor blanked stage (A), nucleate boiling stage (B) and convective cooling stage (C) [45]. Rate of cooling and heat transfer coefficient at similar cooling stages depends on the quenching conditions. Using obtained cooling curves for different quenching conditions several parameters characterizing quenching process were determined: leidenfrost temperature, *T_AB_* (the transition temperature from stage A to stage B), range of cooling rate in stage A, *v_A_*, maximum rate, *v_max_*, temperature at maximum rate, *T_max_*, and maximum value of heat transfer coefficient, *α_max_*. The determined parameters are presented in Table 4.

All the characteristics are credible, with peak values lying in the range reported by other studies [21,22,23,24,25,46]. However, due to the unique mode of hybrid cooling, the results are hard to directly compare. Thus, reliable validation is carried out on two kinds of specimens, as shown in Table 1, a small one—totally encapsulated by the flux, and a big one, the actual forged part, partially covered by the spraying cone at a time. The results are presented in Figure 11a,b, respectively.

Tests with moving probe indicates twice as much as stationary value of HTC at the same conditions. It leads to the conclusion that the sprayer density is not uniform inside the conical stream, which is in agreement with results of related studies [1,23,24,25,26].

To get rid of uncontrollable factors during evaluation of the obtained characteristics of mixed accelerated air and water spray, the first step of validation tests assumed stationary cooling of a simple geometry sample made of steel indicating no diffusion transformation on cooling. Despite justified difficulties with attaining full similarity due to a number of random occurrences, such as exact location, variation of flux in time during experiment, as well as idealized uniformity of air-atomized spray on the surface, impressive consistency between numerical and experimental models was observed (Figure 11a). Equally good agreement between experiment and simulation was concluded for sample 2 (Figure 11b). The numerical modeling results differ where other factors played a role, that is at the very beginning of the test, which is to do with not uniform cooling conditions during transfer (relative movement and difficulties in definition of the beginning of cooling), and at the very end, where consecutive portions of the large sample are gradually leaving the sprayer zone. In fact, the spray exhibits somewhat random distribution, whereas in the numerical model, its domain is defined in a 0–1 manner. Thus, the start and end of cooling in a single point are evident and the same flux density is defined throughout the whole sprayer domain, which results in a slight shifting of the plot towards shorter times.

### 3.3. Analysis of Temperature Evolution during Forging—FEM Modeling

**Figure 12:**
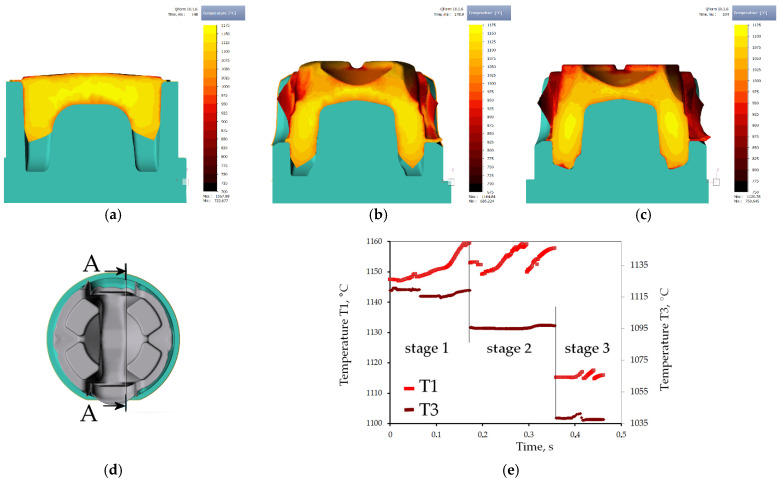
Results of FEM modeling: (**a**–**c**) temperature distribution on the cross-section A-A, (**d**) location of cross-section A-A, (**e**) history of temperature changes during forging chain (excluding feedstock transfer removed for clarity) in locations of thermocouples T1 and T3 (see Figure 1b).

Validation of the HTC estimated for possible settings of cooling elements of the cooling conveyor gives a green light for the essential part of the study—case study on selection of cooling conditions for optimized of structural components and hardness distribution, which are determined by temperature plots in the two crucial locations, T1/T2 and T3/T4 (see Figure 1b), representing areas of different requirements. Contrary to conventional quenching-tempering heat treatment (QT), where soaking temperature is a temperature to start cooling, in thermomechanical controlled processing the run out temperature at start of cooling is resultant from forging process related factors and it is usually varying in the volume. Therefore, possibly an exact estimation of the forge end temperature is needed. Multistage sequence, high temperature and complex path of handling the part between impressions makes thermocouple measurement nearly impossible. Due to large dimensions and the amount of volume to be displaced during deformation, forging was held at high temperatures, which has risen in the aftermath of generation of the deformation heat (Figure 12). The need of free-forge preforming of the billet and the need of rotating with numerous shifting and eventually, trimming is a serious disturbance for thermocouple measurement. Therefore, numerical modeling is the source of temperature evolution to be relied on. In Figure 12a–c, temperature distribution on the surface and in the axial cross-section of the highest temperature (Figure 12d) is shown.

The plotting of temperature tracking in the points of interest through the forging stages is shown in Figure 12. As indicated by the “virtual thermocouples”, the temperature in the points changes asynchronously in relation to one another, starting from 1150 °C, the temperature changes vary between points, due to difference in die chill and heat generation in sections. Finally, during finisher forging, it ranges 1160–1010 °C in volume, and in the point of interest: about 1115 °C in T3 and in T1 approximately 1035 °C. Thus, after deduction of transfer related cooling, 980 °C was assumed for physical modeling.

### 3.4. Optimization of the Conditions of Direct Cooling—Case Study

#### 3.4.1. Physical Modeling

Good agreement of modeling results based on HTC estimated for possible settings of cooling elements of the cooling conveyor gives a green light for the essential part of this work—a case study on selection of cooling conditions for optimized hardness distribution in the two crucial locations (T1/T2 and T3/T4—see Figure 1b), representing areas of different requirements. The cardan coupling ending—the part being considered in the case study—is supposed to indicate high strength in combination with good impact strength and crack resistance in the areas represented by points T1 and T2 (further referred to as “ears”) so as to bear dynamic loads in torsion and bending state of stress. In turn, points T3 and T4 (area referred to as “feet”) are meant to exhibit moderate hardness for easier and economically efficient machining of the grooves on the bottom periphery.

**Figure 13:**
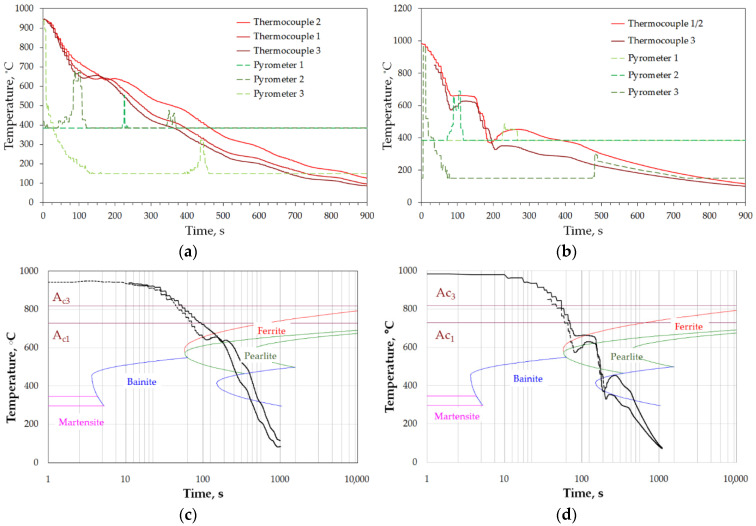
Temperature recorded during experimental tests of cooling with: (**a**) fan accelerated air of 45 m/s, (**b**) air-atomized water spray, and (**c**,**d**) the course of the obtained cooling curves of CTT diagram of steel 38MnVS6 for cooling with accelerated air, and spray, respectively, where: solid lines—T1/T2 and the dashed lines—T3/T4.

For selection of cooling conditions and their expected effect on the properties, cooling trials were modeled, in accordance with Table 2. The results of the physical modeling are presented in Figure 13. As it can be seen from the plots of temperature changes recorded during cooling, there are significant differences between the ears and feet areas. For cooling with fan-accelerated air, these differences are relatively small, with regard to critical points on CTT diagram (Figure 13c), and are mainly caused by time shifts resulting from different times of entering the cooling zone. The small effect of thickness on cooling rate can be explained by the fact of proximity of the ears to inlets. On the contrary, this configuration of sample placement and active inlets makes the flux attenuate while reaching the feet. Hence, despite thicker sections, the cooling rate on the surface of the ears is higher leading to eventual minimization of the difference, whereas while cooling with symmetrically located atomizers, both areas were similarly exposed to action of the spray impingement. Thus, thickness difference plays bigger role in produced cooling rates, which brings about more significant differences in the run of the cooling curves between the two locations on each side (Figure 13b). The difference is reflected by transformations taking place during cooling, as seen from the CTT diagram in Figure 13d).

**Figure 14:**
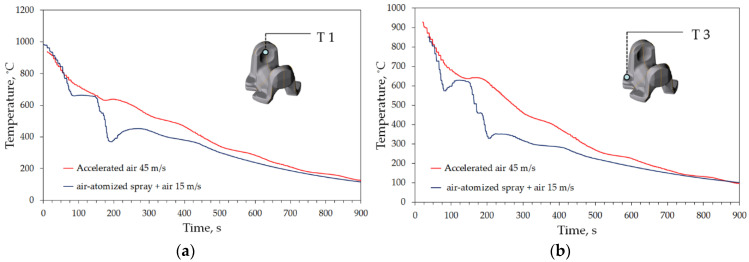
Comparison of the cooling curves of air and spray in locations: (**a**) T1 and (**b**) T3 (see Figure 1c).

For practical reasons of bigger significance is the comparison of the two cooling strategies in one point. As seen in Figure 14, the difference produced by changing cooling medium is significant, reaching 150 °C in points T1 and T2 (ear area), and over 300 °C in the foot area. From the standpoint of operational properties of the forged cardan end, it is desirable to obtain a cooling curve plot similar to that from Figure 14a in points T1 and T2, and the one from Figure 14b in T3 and T4.

#### 3.4.2. FEM Modeling

**Figure 15:**
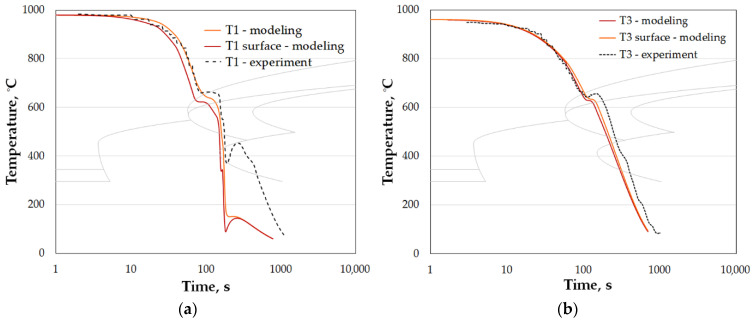
Numerically calculated temperature plots (color lines): (**a**) location T1 (cooling with spray + accelerated air 18 m/s), and (**b**) location T3 (cooling with fan accelerated air 18 m/s); dotted line indicting experimental plot for cooling with the same quenchant alone for previous settings, shown in Table 2.

The obtained cooling plots are shown in Figure 15. One can notice that spray cooling produces larger differences of temperature, which tends to equalize right after spray passes over. A bigger gradient of temperature from the center of the ears towards the surface results in reversed heat flux, causing the temperature increase, reaching 100 °C (decreasing towards the center of the cross-section). Its presence on the numerically estimated curves and its correct location on CCT diagram indicates reliability of the transformation model.

**Figure 16:**
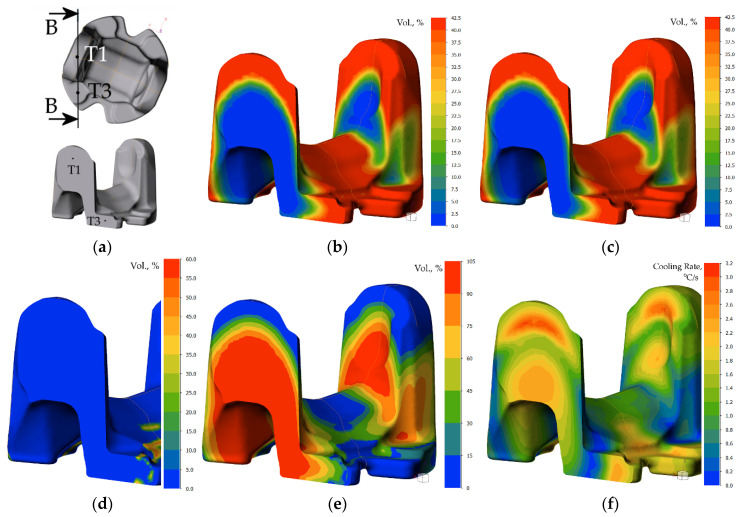
Numerical modeling results for time 60 s (after sprayer1): (**a**) location of cross-section in plane of points T1 and T3, and calculated volume fractions of: (**b**) ferrite, (**c**) pearlite, (**d**) bainite, and (**e**) austenite (untransformed). (**f**) Instant cooling rate.

**Figure 17:**
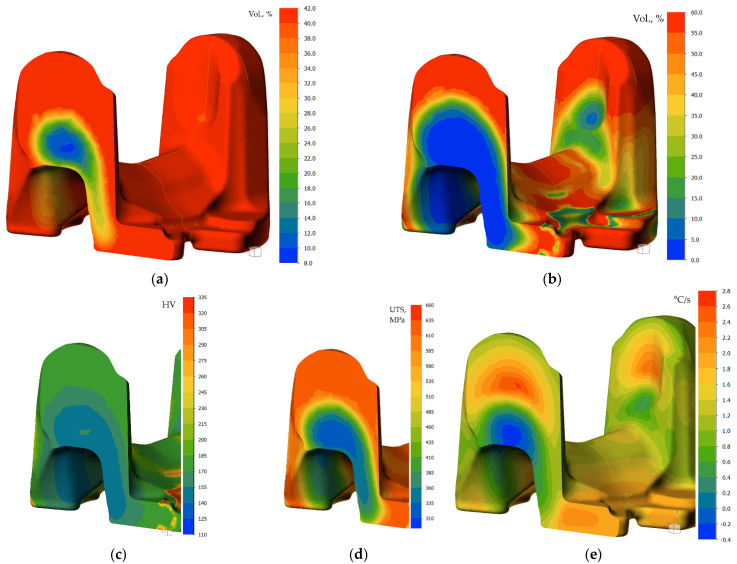
Numerically calculated volume fractions of: (**a**) ferrite, and (**b**) pearlite, and (**c**) hardness, (**d**) ultimate tensile strength, and (**e**) instant cooling rate for cooling time 100 s—after sprayer 2.

**Figure 18:**
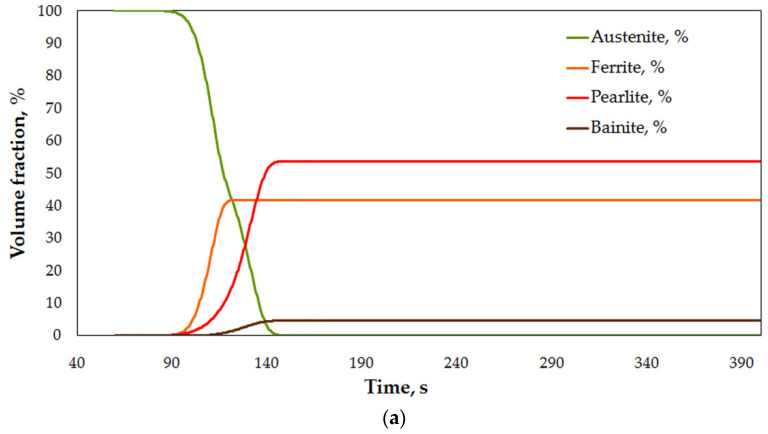

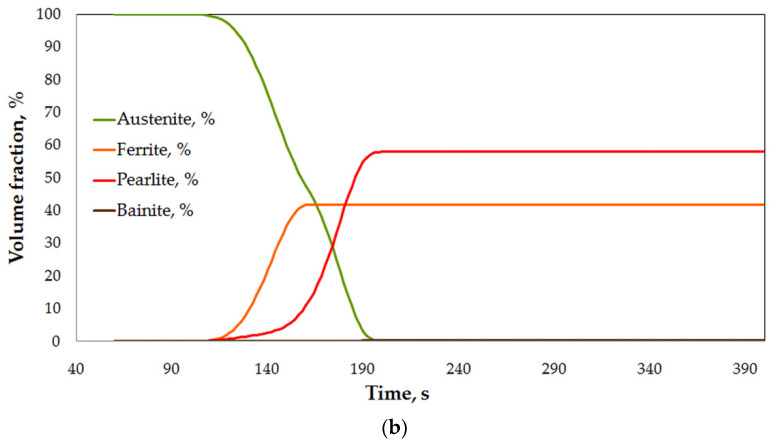
Numerically calculated volume fractions in locations: (**a**) T1 (spray), and (**b**) T3 (fan).

Analysis of the cooling plots from physical modeling in the critical points suggests combining the two cooling cycles with local differentiation of cooling rate. Having met the minimum of required hardness in the whole volume (including the thickest cross-section), attempt was made to decrease the cooling rate in the volume by local enhancement of cooling rate in the ear area by use of water spray (in addition to accelerated air blow in the cooling zone). Thus, heat transfer coefficient function for setting 5 was assumed in domains, among an environment with a defined heat transfer coefficient corresponding to air rate 18 m/s [31]. The results of this effort are shown in Figure 16, Figure 17 and Figure 18.

Elucidation of progress of the transformation of austenite on cooling shows evident differentiation of cooling conditions, which results in a higher cooling rate in the upper portion of the heat treated part, and eventually, higher hardness and predicted strength, whose onset can be noticed after passing the second atomizer. The predicted structural composition of the microstructure after cooling is ferrite over 42% and pearlite (about 58%), which decreases in the location T1 and T2, giving way to 5.7% bainite precipitation, resulting in higher strength, finally reaching 840 MPa.

The full scope of microstructure changes during cooling is illustrated graphically in Figure 18. The graph confirms a faster onset of precipitation of austenite decomposition products, and a higher amount of bainite. The results of modeling confirm assumptions as to the utility of local enhancement of cooling for forged parts to control the kinetics of transformation during direct quenching of steel. Obviously, the variation of cooling conditions has its limits. Due to close vicinity of sprayed zone, the conductive mechanism of heat transfer, as well as significant surface of emission of the heat, the cooling times observed for accelerated air in feet areas are shorter than those of spray-cooled ears, despite the difference in section thickness. In this light, the importance of total heat capacity of the heated/cooled volume must be taken into account.

## 4. Discussion

**Figure 19:**
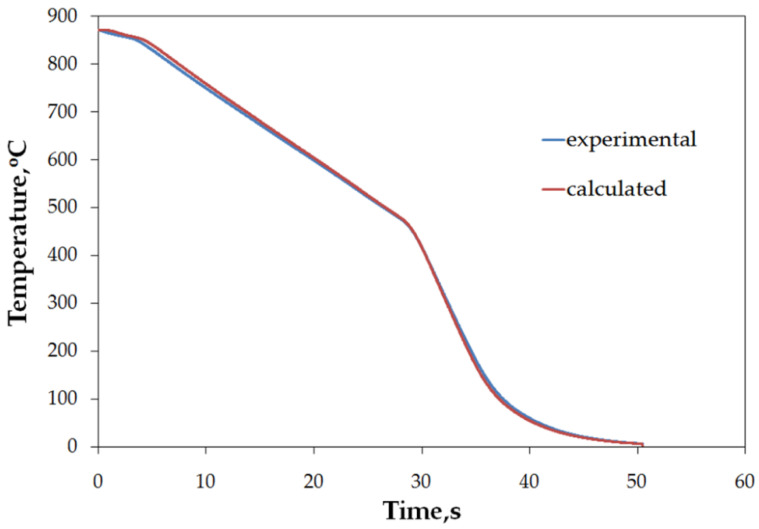
Comparison of experimental and calculated temperature inside of testing probe for quenching according designation setting 1.

In the processes of heat treatment of metals and forecasting structural effects of phase transformations of undercooled austenite, the knowledge of the characteristics of the cooling capacity of the cooling medium plays an important role. This characteristic is the dependence of the heat transfer coefficient, *α*, on the surface temperature, *T_p_*, of the cooled metal object. The calculation of this dependence is based on the experimental data obtained by recording the temperature with a thermocouple placed in the center of the probe with a specific size and geometric shape, heated to a specific temperature and placed in a cooling medium. There are a number of methods for measuring the cooling capacity of quenching media [45]. One of them is the method, in which the probe is a cylinder constructed from Inconel 600 with dimensions: diameter, d = 12.5 mm, height: h = 60 mm, with a thermocouple placed in the center [47]. The obtained cooling curve (dependence of the temperature of the center of the probe on cooling time) is used to calculate the dependence of the heat transfer coefficient, α, on the surface temperature, *T_p_*, using the heat conduction equation. The obtained temperature data contain disturbances caused by the occurrence of electronic noise during the measurements. Selected computer programs, for example MatLab, can be used to eliminate them. Then, by solving the modified heat conduction Equation (1), the surface temperature, *T_p_*, of the test probe, the heat flux, *q*, and the heat transfer coefficient, *α*, are calculated. The calculated heat transfer coefficient includes the effect of all physical phenomena occurring on the surface of the cooled object in contact with the cooling medium during successive stages of quenching process. The properties of the cooling medium, its ability to dissipate heat from metallic object, have a decisive influence on the value of the heat transfer coefficient. A measure of the accuracy of calculating the dependence of the heat transfer coefficient as a function of surface temperature can be the comparison of the calculated (using the calculated dependence *α = f (T_p_)*) temperature inside the probe with the temperature obtained experimentally. Comparison of calculated and experimental temperature in the middle of the probe for designation heat treatment setting 1 is shown in Figure 19.

As can be seen, the compatibility is very good. Therefore, the use of the calculated dependence (*α = f (T_p_)*) for practical calculations of field temperature under heat treatment conditions identical to those for measuring the cooling ability of applied medium should not introduce any additional errors. However, it should be mentioned that the value of the current heat transfer coefficient is significantly influenced by the oxidation state of the surface of the cooled metallic object as well as the rate of movement of the cooling medium. Calculated parameters (*T_AB_*, *v_A_*, *v_max_*, *T_max_*, *α_max_*) and relationships *α = f(T)*, characterizing the efficiency of heat transfer from testing probe quenched at different conditions showed a significant effect of conditions on these parameters during different stages of quenching process.

The presented results confirm correctness of the models based on employing mobile domains of defined heat transfer characteristics and accuracy of the heat transfer coefficient estimation, based on solution of inverse problem. The final solution of the technological issues, where the calculated fractions of structural components and hardness are ultimate are adversary of the credibility of the results. Good agreement of the numerically calculated and measured plots of temperature in time of cooling for complex geometry allows for versatile use of the models in comprehensive analysis of thermomechanical processing of a variety of steel products.

Hot forging of die-forged cardan, a complex-geometry part weighing nearly 12 kg with large surface/weight ratio and significant differences of cross-sections, is a good example for a case study on modeling of hot forging and subsequent controlled cooling, where local variation of cooling rate is sought for [48]. Due to significant differences in wall thickness (cross-sections), natural variations in cooling rate occur on cooling, causing serious problems in maintaining optimized hardness, on one hand, and ductility, on the other hand. As the leading requirement is the combination of mechanical properties, machining of the part must be done at the expense of excessive tool wear. Therefore, the most favorable distribution of cooling rate in time needs overcoming natural flow of the cooling curves in locations being subject to inspection. One of the methods to use in this aspect is application localized enhancement of cooling intensity with use of water spray. However, number of parameters controlling the jet impingement effects make it hard to reproduce results of the laboratory test, especially, those conducted in stationary conditions into industrial manufacturing lines. Numerical modeling can offer an aid in prediction of the effects of given parameters of jet impingement on the cooling curves plots in relation to transformation points of a given steel grade, thereby indicating a favorable combination for experimental testing (or the wrong ones to drop out), saving time and costs.

Sound input data and boundary conditions in modeling are some of the conditions for results reliability. The presented study highlights the difficulties one has to cope with while modeling interrupted cooling with use of diversified cooling environments, including mixed sprayed with accelerated air and/or movement of the part being cooled, with assumption of proper model of simultaneous action of multiple environments and acquisition of reliable characteristics of cooling media in interrupted cooling at the forefront. In this respect, estimation of heat transfer coefficient is crucial. Of great significance is the manner in which they are implemented in FEM simulation to provide similarity of air/spray flow around an object being cooled.

The presented results confirm correctness of the models based on employing mobile domains of defined heat transfer characteristics and accuracy of the heat transfer coefficient estimation, based on solution of inverse problem, used in determination of HTC functions. The final solution of the technological issues, where the calculated fractions of structural components and hardness are ultimate adversary of the credibility of the results. Good agreement of the numerically calculated plots of temperature in time with those experimentally measured during cooling a complex-geometry forged part allows for versatile use of the models in comprehensive analysis of thermomechanical processing of a variety of steel products.

## 5. Conclusions

Application of water spray cooling gives wide possibilities of creation of cooling strategies for desired effects of transformation kinetics during thermomechanical treatment. As shown in the presented study, the use of air-atomized water sprays allows for local enhancement of the cooling rate to influence the heat flux in the volume of complex geometries.

Numerical methods based on the solution of inverse problems supports estimation of heat transfer coefficient to compose numerical models for FEM simulation of controlled cooling for mixed environments found in industrial cooling conveyors equipped with atomizers, which may be hard for mathematical description. The use of FEM modeling for design of controlled cooling cycles of parts with varied cross-sections and wall thickness in the volume calls for attaining similarity in flow dynamics of the cooling media. Application of domains for specification of unique cooling conditions helps in creation of reliable boundary conditions for reliability of the numerical model.

Suitability of the presented approach for selection of process conditions in designing of thermomechanical treatment of steel without engagement of production assemblies is shown on the example of a hot forged cardan end, for which the model enabled successfully determine diversified conditions for reversal of heat flux caused by the natural cooling tendency to produce required distribution of microstructural composition and mechanical properties. Thus, the presented study may be of utilitarian use for similar cases of geometry and microstructure-property combinations.

## Figures and Tables

**Table 1:** The cooling conditions selected for measurement of the heat transfer coefficient.

Designation	Cooling Media	Location of Atomizers	Conveyor Speed, m/min	Flow Efficiency		Configuration Scheme
				Air, m/s	Water, Bar	
settings 1	spray	1	off	-	1.8–1.95	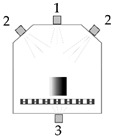
settings 2	spray	1 + 3	off	-	1.8–1.95	
settings 3	spray + air	1	off	18	1.96	
settings 4	spray + air	2 + 2	off	18	1.75	
settings 5	spray + air	1 → 2	0.4	25	2.08	

**Table 2:** The cooling conditions assumed for the case study cooling tests.

Test	Cooling Media	Air Rate, m/s	Conveyor Speed
			m/min
test 1	Fan-accelerated air only	45	0.4
test 2	Fan-acc. air + water spray	12–15	0.4
test 3	Fan-acc. air (T3/T4) ^1^, water spray (T1/T2) ^1^	12–15	0.6

^1^ See Figure 1b for reference.

**Table 3:** Flow stress model parameters of steel 38MnSV6 used in simulation of forging.

Parameter	A	*m*_1_	*m*_2_	*m*_3_	*m*_4_	*m*_5_	*m*_7_	*m*_8_	*m*_9_
Value	5209.8	−0.0031	0.4236	−0.015	0.00043	−0.0006	−0.3316	0.00014	0.0

**Table 4:** Characteristic parameters of quenching processes.

Designation	*T_AB_*	*v_A_*	*v_max_*	*T_max_*	*α_max_*
	°C	°C/s	°C/s	°C	W/(m^2^ °C)
settings 1	480	14 ÷ 16	48.3	350	4750
settings 2	463	10 ÷ 12.8	39	305	3500
settings 3	360	7 ÷ 11	33.5	200	4116
settings 4	445	13.8 ÷ 15.8	56.5	346	6050
settings 5	440	12 ÷ 21	88.3	330.5	18,062

## Data Availability

Additional data can be shared on request.

## Nomenclature

*a*	thermal diffusion coefficient
*A*	Hensel-Spittel equation coefficient
*c_p_*	specific heat
*d*	diameter of test probe
*e*	Euler’s number
*K*	number of time steps
*m*	Hensel-Spittel equation coefficients
*n_p_*	number of grid space
*q*	heat flux
*r*	distance from the axis of the specimen
*R*	radius of test probe
*R^2^*	correlation coefficient
*T*	temperature
*t*	time
*T_AB_*	the transition temperature from stage A to stage B
*T_measured_*	measured temperature
*T_p_*	surface temperature
*v*	cooling rate
*v_A_*	cooling rate in stage A
**Subscripts:**	
*i*	index of space point
*max*	maximum value
**Superscripts:**	
*k*	index of time point
**Greek symbols:**	
*α*	heat transfer coefficient
*β*	non-negative coefficient
*ε*	strain
$\dot{\varepsilon }$	strain rate
Δ*t*	time interval
Δ*r*	radius interval
*λ*	thermal conductivity
*ρ*	density
*σ_p_*	flow stress
*τ*	time
